# Comparisons of Robustness and Sensitivity between Cancer and Normal Cells by Microarray Data

**DOI:** 10.4137/cin.s386

**Published:** 2008-03-28

**Authors:** Liang-Hui Chu, Bor-Sen Chen

**Affiliations:** Lab of Control and Systems Biology, National Tsing Hua University, Hsinchu, 300, Taiwan

**Keywords:** robustness tradeoffs, sensitivity analysis, robustness-based cancer drug design, feedback loops of p53

## Abstract

Robustness is defined as the ability to uphold performance in face of perturbations and uncertainties, and sensitivity is a measure of the system deviations generated by perturbations to the system. While cancer appears as a robust but fragile system, few computational and quantitative evidences demonstrate robustness tradeoffs in cancer. Microarrays have been widely applied to decipher gene expression signatures in human cancer research, and quantification of global gene expression profiles facilitates precise prediction and modeling of cancer in systems biology. We provide several efficient computational methods based on system and control theory to compare robustness and sensitivity between cancer and normal cells by microarray data. Measurement of robustness and sensitivity by linear stochastic model is introduced in this study, which shows oscillations in feedback loops of p53 and demonstrates robustness tradeoffs that cancer is a robust system with some extreme fragilities. In addition, we measure sensitivity of gene expression to perturbations in other gene expression and kinetic parameters, discuss nonlinear effects in feedback loops of p53 and extend our method to robustness-based cancer drug design.

## Introduction

Robustness is a measure of the system behavior or performance in face of perturbations and uncertainties, and sensitivity is a measure of the system deviations generated by perturbations to the system, for instance, environmental stress (Chen et al. 2005; Stelling et al. 2004; Weinmann, 1991). Since robustness or sensitivity is an essential property of biological systems, the general concept has been proposed recently (Kitano, 2004a; Stelling et al. 2004), and several analytical methods of robustness and sensitivity have been recently developed (Chen et al. 2005; Sontag et al. 2004). Many studies discuss robustness and sensitivity from theories to experiments, for example, researchers design robust bacterial signaling networks based on the control theory to adapt to the environment (Kollmann et al. 2005), and some studies design filters to regulate variations and preserve robustness (Chen and Wang, 2006).

Cancer is a complex system caused by multiple gene mutations including oncogenes and tumor-suppressor genes (Vogelstein and Kinzler, 2004). Tumors preserve cellular diversity which facilitates robustness through homogeneous and heterogeneous redundancies and feedback loops (Kitano, 2004b). For example, transcriptional activation of the tumor suppressor gene p53 produces several positive and negative autoregulatory feedback loops that interact with many other signal transduction pathways (Harris and Levine, 2005). Multiple feedback loops provide redundancies, strengthen robustness and reduce phenotype variations (Stelling et al. 2004). Tumors are highly robust to maintain their proliferation potential against a wide range of anticancer therapies, which are exploited by robustness-functional redundancy and feedback-control systems (Kitano, 2004b). Multiple feedback loops of p53, for example, can prevent errors and reduce the phenotype of mutations (Harris and Levine, 2005).

Definition of sensitivity in systems control theory is the reciprocal of robustness (Chen et al. 2005; Voit and Ferreira, 2000), which can help designers evaluate perturbated gene expression by environmental stress. Cancer displays the ‘robust yet fragile’ property, that is, robustness tradeoffs, because cancer gains robust abilities to anti-growth signals but hypersensitive functions to tissue invasion (Kitano, 2004b). The tradeoffs among robustness, fragility, and complexity dominate design procedure of many engineering systems, and now the theoretical foundation for a systems-oriented drug design has been recently developed. A drug target can be effective if it hits the point of fragility, and tradeoffs among robustness, fragility, and complexity dominate the theoretical foundation for a systems-oriented drug design (Carlson and Doyle, 2002; Chen and Li, 2007; Kitano, 2004b, 2007). While robustness tradeoffs in biological organisms have been discussed recently and control theories have been proposed for a long time, quantitative confirmation of robustness by system control theory in cancer research is necessary.

Through the progression of the large-scale experiment, the complexity and multiple factors in cancer cells seem feasible to detect and measure. The large-scale sequencing approach such as microarray technology identifies several previous unknown mutations, giving us whole genome expression profiles to detect gene expressions and target genes. Most computational analysis of the microarray data relies on clustering which broadly classifies genes’ functions into subgroups but hides many possible transcription factors (Barenco et al. 2006). Moreover, the clustering approach cannot describe systematic behavior of the gene regulatory circuits such as robustness and sensitivity (Sontag et al. 2004). Mathematical modeling, on the contrary, could sketch the wiring diagrams of cellular signaling pathways in this ‘integrated circuit of the cell’ (Hanahan and Weinberg, 2000).

The systems biology approach by microarray data and mathematical models is powerful to measure robustness and sensitivity in cancer cells, one of the most important issues in robustness-based drug target design (Hornberg et al. 2006; Kitano, 2007). Although the concept of robustness tradeoffs has been proposed (Kitano, 2004a, b, 2007; Stelling et al. 2004), few studies calculate robustness and sensitivity in cancer quantitatively. In this study, we use linear discrete stochastic model from microarray data to analyze robustness and sensitivity in feedback loops of p53 by system control theory and extend our results to robustness-based cancer drug design.

## Methods

### Selecting and processing experimental data

The p53 tumor suppressor gene is described as ‘the guardian of the genome’, which prevents mutagenesis and carcinogenesis by promoting cell-cycle arrest or apoptosis (Vogelstein and Kinzler, 2004; Vousden and Lu, 2002). Therefore, we choose p53 as our example to display robustness properties in cancer cells. Microarray data is collected from Barenco et al. (2006), in which human leukemia cell lines (MOTL4) containing functional p53 are irradiated at 0, 2, 4, 6, 8, 10, 12 hours in triplicate. Due to multiple feedback loops of p53 that determine systematic robust behavior (Kitano, 2004b), genes of the multiple positive or negative feedback networks are primarily considered in our analysis of robustness of p53.

To inquire into systematical analysis of sensitivity under diverse stresses upon the p53 system, we apply the microarray data of HeLa cervical carcinoma cells and primary human lung fibroblasts to heat shock, oxidative stress, and endoplasmic reticulum (ER) stress (Murray et al. 2004). We induced heat shock by transferring culture flasks from a 37 °C incubator to a 42 °C incubator. ER stress was induced by treating HeLa cells or fibroblasts with 2.5 mM dithiothreitol (DTT). Oxidative stress was induced by treating HeLa cells or fibroblasts with 10 μM menadione bisulfate. Time points are sampled as 0, 0.5, 1, 2, 3, 4, 6, 8, 24 hours in cancer cells and 0, 0.5, 1, 2, 4, 10, 16, 24 hours in normal cells under heat stress, compared to 0, 0.5, 1, 2, 4, 8, 12, 24 hours in cancer cells and 0, 0.5, 1, 3, 4, 6, 8, 12, 24 hours in normal cells under oxidative stress, and 0, 0.5, 1, 2, 4, 6, 8, 16, 24 in cancer cells and 0, 0.5, 1, 2, 3, 4, 6, 8, 12, 16, 24 in normal cells under ER stress.

### Robustness analysis in feedback loops of p53

Assume the gene regulatory network contains *M* genes, and time-series microarray data contains *N* time samplings. The system is modeled as:

(1) $$\bar{x}[t+1]=A\bar{x}[t]+\bar{k}+\bar{ɛ}[t]$$

where *x̄*[*t*]=[*x*_1_ (*t*) *x_2_* (*t*) … *x_M_* (*t*)]*^T^* denotes mRNA levels of total *M* genes at time point *t* = 1 … *N. k̄* stands for the basal level from other intrinsic and extrinsic pathways, and *ɛ̄*[*t*] represents stochastic events such as modeling errors, intrinsic and extrinsic noises (Chen and Wang, 2006). We use the least square estimation method to estimate the interaction matrix *A* in equation (1) from time-series microarray data as

(2) $$A=\left[\begin{array}{cccc} \alpha_{1,1} & \alpha_{1,2} & \ldots & \alpha_{1,M}\\ \alpha_{2,1} & \ddots & & \alpha_{2,M}\\ \vdots & & \ddots & \\ \alpha_{M,1} & \alpha_{M,2} & & \alpha_{M,M} \end{array}\right]$$

where *α_i,j_* denotes the interaction from gene *j* to gene *i* when *i* ≠ *j*, ∀ *i*, *j* = 1 … *M*.

Based on system theory (Weinmann, 1991), the eigenvalues *λ_i_, i* = 1 … *M* in equation (2) determine the stability of the network. If eigenvalues of *A* are all inside the unit circle of the z-complex domain (that is, |*λ_i_*| < 1), the network is stable; if at least one eigenvalue is outside the unit circle (that is, |*λ_i_*| > 1), the network is unstable; if some eigenvalues are on the unit circle (that is, |*λ_i_*| = 1), the network will be oscillated. If all eigenvalues are more near the origin of the z complex domain, the network is more robust because its eigenvalues are not easily perturbed outside the unit circle.

### Sensitivity analysis of cancer and normal cells under stresses

Sensitivity is the reciprocal of robustness which measures the system response to external disturbance (Voit and Ferreira, 2000). In this study, we introduce three methods to measure sensitivity in different meanings: sensitivity of perturbations in gene expression to external stress, sensitivity of gene expression to the perturbations in other gene expression, and sensitivity of gene expression to changes in kinetic parameters.

We can describe a system with external disturbances as

(3) $$\bar{x}[t+1]=A\bar{x}[t]+B\bar{u}[t-1]+\bar{k}+\bar{ɛ}$$

where *x̄*[*t*] denotes the gene expression profiles at time *t* measured in the microarray, *ū*[*t* − 1] denotes an environmental stress on the system at time *t* − 1 with one time delay, *k̄* represents the basal level from other unconsidered influence, and *ɛ̄*[*t*] represents stochastic events such as intrinsic and extrinsic noises. We also apply the least square estimation algorithm (Johansson, 1993) to obtain parameters of *A* and *B* by microarray data. *ū*[*t* − 1] is considered as a step function which represents constant environmental stress to the gene regulatory circuits with time delay in response.

### Sensitivity of the gene expression for perturbations in environmental stress

The sensitivity for the input stress could be derived as

(4) $$S(t)≜\frac{△\bar{x}[t+1]}{△\bar{u}[t-1]}=A\frac{△\bar{x}[t]}{△\bar{x}[t+1]}•\frac{△\bar{x}[t+1]}{△\bar{u}[t-1]}+B$$

where Δ means a small perturbation in discrete time system, and $\frac{△\bar{u}[t-1]}{△\bar{u}[t-1]}=I,\frac{△\bar{k}}{△\bar{u}[t-1]}\;=0$, and $\frac{△\bar{ɛ}}{△\bar{u}[t-1]}=0$. Therefore, we can derive the sensitivity as

(5) $$S(t)≜\frac{△\bar{x}[t+1]}{△\bar{u}[t-1]}={\left(I-A\frac{△\bar{x}[t]}{△\bar{x}[t+1]}\right)}^{-1}B$$

In equation (5), *S*(*t*) is time-dependent function which reflects the sensitivity to perturbations at different time samplings, which represent the system variations to unit-step input perturbations. For clarity and simplicity in comparison with sensitivity scores of *M* genes in cancer cells with those in normal cells, we define the sensitivity score of each gene as the average of equation (5)

(6) $$\begin{array}{l} S_{i}=\text{Sensitivity score of gene }i\\ ≜\frac{1}{N}\sum_{t=1}^{N}|\frac{△x_{i}[t+1]}{△u_{i}[t-1]}|\;i=1\ldots M \end{array}$$

### Sensitivity of gene expression to the perturbations in other gene expression

Besides sensitivity analysis in equations (3)–(6) that measure changes in a system state with respect for environmental perturbations, the relative change of a system variable in response to a relative change in the other system variables is also important in analyzing sensitivity and robustness (Voit and Ferreira, 2000). In other words, measurement of △;*x̄*[*t* + 1] to △*x̄*[*t*] can represent stability of the system. Since $\frac{△\bar{k}}{△\bar{x}[t]}=0$ and $\frac{△\bar{ɛ}}{△\bar{x}[t]}=0$, we gain sensitivity of the perturbated gene expression from equation (3) as

(7) $$S(t)≜\frac{△\bar{x}[t+1]}{△\bar{x}[t]}=A+B\frac{△\bar{u}[t-1]}{△\bar{x}[t+1]}•\frac{△\bar{x}[t+1]}{△\bar{x}[t]}$$

Therefore, sensitivity of the perturbated gene expressions is

(8) $$S(t)≜\frac{△\bar{x}[t+1]}{△\bar{x}[t]}={\left(I-B\frac{△\bar{u}[t-1]}{△\bar{x}[t+1]}\right)}^{-1}A$$

In general, $\frac{△\bar{u}[t-1]}{△\bar{x}[t+1]}=0$ because the control input *ū*[*t* − 1] is independent of state vector *x̄*[*t* + 1] except the control input is based on state feedback. In this situation, $\frac{△\bar{u}[t-1]}{△\bar{x}[t+1]}=0$ because perturbation of gene expression *x̄*[*t* + 1] should be independent of control input as extracellular stress *ū*[*t* − 1]. So, we approximate equation (8) as

(9) $$S(t)≜\frac{△\bar{x}[t+1]}{△\bar{x}[t]}=A$$

It is noticed *S*(*t*) is approximate to *A* in equation (3) if we consider $\frac{△\bar{u}[t-1]}{△\bar{x}[t+1]}=0$. Hence, *A* primarily determines stability and robustness of the system, which coordinates with robustness analysis in equations (1)–(2). If all the eigenvalues *λ_i_* of *A* are inside the unit circle in z-complex domain, the propagation of perturbations of concentration of genes will converge to zero constant. On the other hand, if some eigenvalues *λ_i_* of *A* are outside the unit circle in z-complex domain, perturbations in the concentrations of some genes will deteriorate or amplify in the gene network. If some eigenvalues of the network are near the unit circle, then the perturbation of some gene units will keep propagation in the gene circuit.

### Sensitivity of gene expression to perturbations of parameter *A*

In this method, sensitivity is used to estimate the effect of variations of parameters *A* in equation (1). However, we can not directly calculate $\frac{△\bar{x}[t+1]}{△A}$ because *A* is a matrix. Therefore we calculate $\frac{△\bar{x}[t+1]}{△A}$ within *M* genes from equation (3) by the definition in Weinmann (1991).

(10) $$\begin{array}{l} \frac{△\bar{x}[t+1]}{△A}≜\sum_{i,j}E_{i,j}⊗\frac{△\bar{x}[t+1]}{△A_{i,j}}\\ =\left[\begin{array}{cccc} \frac{△\bar{x}[t+1]}{△A_{1,1}} & \frac{△\bar{x}[t+1]}{△A_{1,2}} & \cdots & \frac{△\bar{x}[t+1]}{△A_{1,M}}\\ \frac{△\bar{x}[t+1]}{△A_{2,1}} & \frac{△\bar{x}[t+1]}{△A_{2,2}} & \cdots & \frac{△\bar{x}[t+1]}{△A_{2,M}}\\ \vdots & \vdots & \ddots & \vdots \\ \frac{△\bar{x}[t+1]}{△A_{M,1}} & \frac{△\bar{x}[t+1]}{△A_{M,2}} & \cdots & \frac{△\bar{x}[t+1]}{△A_{M,M}} \end{array}\right]\\ =\;\text{matrix}\left[\frac{△\bar{x}[t+1]}{△A_{i,j}}\right],\forall i,j=1\ldots M \end{array}$$

In equation (10), ⊗ denotes Kronecker product and *E_i,j_* denotes Kronecker matrix (Weinmann, 1991). By matrix product rule, we can derive equation (10) from equation (3) as

(11) $$\begin{array}{l} \frac{△\bar{x}[t+1]}{△A}=\frac{△(A\bar{x}[t])}{△A}=\sum_{i,j}E_{i,j}⊗\frac{△(A\bar{x}[t])}{△A_{i,j}}\\ =\sum_{i,j}\left(E_{i,j}⊗\frac{△A}{△A_{i,j}}\bar{x}[t]+E_{i,j}⊗A\frac{△\bar{x}[t]}{△A_{i,j}}\right)\\ =\sum_{i,j}E_{i,j}I⊗\frac{△A}{△A_{i,j}}\bar{x}[t]\\ +\sum_{i,j}\left(IE_{i,j}⊗A\frac{△\bar{x}[t]}{△A_{i,j}}\right)\\ =\sum_{i,j}\left(E_{i,j}⊗\frac{△A}{△A_{i,j}}\right)(I⊗\bar{x}[t])\\ +\sum_{i,j}(I⊗A)\left(E_{i,j}⊗\frac{△\bar{x}[t]}{△A_{i,j}}\right) \end{array}$$

For simplicity in comparison with the sensitivity of gene expression to each △*A_i,j_*_,_ ∀ *i*,*j* = 1 … *M*, we define the sensitivity score *S_i,j_* of each △*A_i_*_,_*_j_* as the average of equation (10) or (11), that is,

(12) $$\begin{array}{c} S_{i,j}=\text{Sensitivity scores of perturbated }A_{i,j}\\ ≜\frac{1}{N}\sum_{t=1}^{N}|\frac{△\bar{x}[t+1]}{△A_{i,j}}|\forall i,j=1\ldots M \end{array}$$

All the simulations are with MATLAB 7.1, and the programs can be requested by email to the authors. Cubic spline interpolation is also used in the simulation to obtain sufficient time samplings in the silico simulation and parameter estimation without data overfitting or deviating data (Chang et al. 2005).

## Results

### Construction of multiple feedback loops of p53 pathway

**Figure 1:**
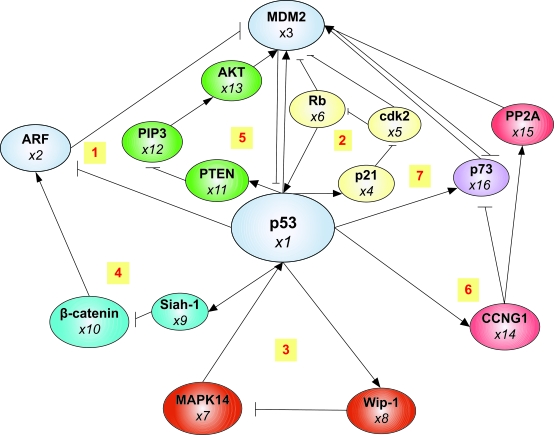
Multiple feedback loops of p53 pathway. Several positive, negative and autoregulatory feedback loops are illustrated. These sixteen genes in p53 pathway are: p53, ARF, MDM2, p21, cdk2, Rb, MAPK14, Wip-1, Siah-1, β-catenin, PTEN, PIP3, AKT2, CCNG1, PP2A, and p73.

The P53 protein and its signal transduction pathway are composed of a set of genes and their protein products that are designed to respond to a wide variety of intrinsic and extrinsic stress signals (Vogelstein and Kinzler, 2004). We combine several literature to construct p53 feedback regulatory network within seven feedback loops (Fodde et al. 2001; Harris and Levine, 2005; Sherr, 2006; Sherr and McCormick, 2002; Vivanco and Sawyers, 2002; Vousden and Lu, 2002) including sixteen genes, namely p53, ARF, MDM2, p21, cdk2, Rb, MAPK14, Wip-1, Siah-1, β-catenin, PTEN, PIP3, AKT2, CCNG1, PP2A, and p73 (shown in Fig. 1).

#### Loop1

p53/MDM2/ARF. p53 transcriptionally activates MDM2, while activated MDM2 targets p53 by both inhibiting its activity as a transcription factor and enhancing its degradation rate (p53 → MDM2 –| p53). The concentration of p53 increases in response to stress signals such as DNA damage, while different stress signals undergo distinct pathways to allow p53 to escape MDM2-mediated protein degradation (Harris and Levine, 2005; Toledo and Wahl, 2006). The ARF tumor suppressor gene plays an important role in cell cycle regulation or apoptosis by controlling MDM2 and p53 levels (ARF –| MDM2 –| p53). Transcription of the ARF gene is positively regulated by β-catenin and negatively regulated by p53 itself, and ARF protein binds to the MDM2 protein and increase the level of P53 protein (Sherr, 2006).

#### Loop2

p21/Cyclin E-cdk2/Rb/MDM2. The p53 transcription factor is induced in response to DNA damage and oncogene activation, and p53 regulates a set of genes either related to cell cycle arrest or apoptosis such as p21. p21 and p27 are the Cip/Kip family of cyclin-dependent kinase inhibitor that inhibits cyclin E-cdk2 complex, which phosphorylates and inhibits both Rb and MDM2 (Sherr and McCormick, 2002). Rb directly binds to MDM2 to antagonize these functions and then inhibits p53 activity.

#### Loop3

Wip-1/MAPK14. Wip-1 is a p53 responsive gene forming a negative autoregulatory loop connecting the p53 and Ras pathways, which also dephosphorylates and inactivates MAPK14 (p38MAP kinase). MAPK14 is also regulated through the Ras signaling cascade and mediates phosphorylation of p53 in response to UV irradiation (Vousden and Lu, 2002).

#### Loop4

Siah1/β-catenin/ARF. The activated P53 protein positively regulates the transcription of the ubiquitin ligase Siah-1 which in turn acts to degrade the β-catenin protein. β-catenin can regulate ARF which in turn negatively regulates MDM2 and results in higher p53 levels (Fodde et al. 2001).

#### Loop5

PTEN/AKT2/MDM2. In some cell types, p53 can positively regulate PTEN, a lipid phosphatase that converts PIP3 to PIP2, and then PIP3 activates AKT2 kinase which influences cell survival by phosphorylation of MDM2 resulting in translocation of MDM2 into the nucleus and inactivation of p53. This connection forms a positive feedback loop for enhancing p53 activity and decreasing AKT2 activity (Vivanco and Sawyers, 2002).

#### Loop6

CCNG1/MDM2. One of the p53-responsive genes includes cyclin G protein (CCNG1), forming a complex with the PP2A phosphatase which removes a phosphate residue from MDM2. The cyclin G-PP2A phosphatase enhances MDM2 activity and inhibits p53 (Sherr, 2006).

#### Loop 7

p73/p53-regulated genes. p53 is activated by environmental stresses and in turn stimulates the transcription of a particular spliced mRNA from the p73 gene named p73 ΔN. When p53 activates the transcription of p73 ΔN, the p73 ΔN protein can bind many of the p53-regulated genes, but the absence of a transactivation domain makes it act as a repressor or competitor of p53 transcriptional activation. In this way, a negative feedback loop is set up and p53 activity declines (Harris and Levine, 2005).

### Oscillations in feedback loops of p53 pathway

**Figure 2:**
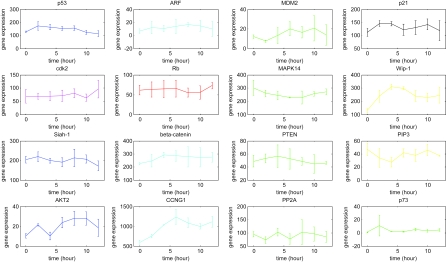
Mean and standard deviation of gene expressions of sixteen genes in multiple feedback loops of p53.

We construct feedback loops of p53 with sixteen genes in Figure 1 by literature and plot means and standard deviations of the triplicate discrete normalized microarray data in Figure 2. By least square error parameter estimation (Johansson, 1993), the dynamic model of the sixteen genes in the p53 feedback network in equation (1) is constructed in Table 1 of sixteen genes with estimated parameters by microarray data (Barenco et al. 2006). Through equations (1)–(2) and Table 1, we calculate sixteen eigenvalues of interaction matrix *A* as follows:

0.755350.937620.941230.95820.967920.977020.970470.998481.01211.01560.94887+ 0.052618i0.94887 − 0.052618i0.98245 + 0.0033062i0.98245 − 0.0033062i1.0516 + 0.023984i1.0516 − 0.023984i

**Figure 3:**
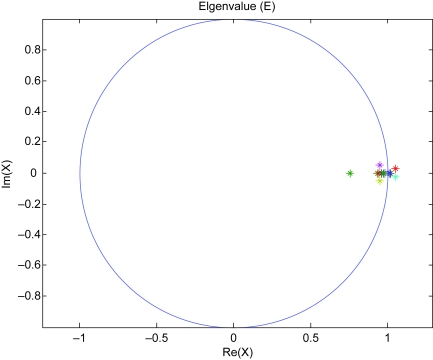
Plot of the sixteen eigenvalues in multiple feedback loops of p53. Fifteen eigenvalues are located near the unit circle, strongly indicating that p53 system is oscillated.

The result in Figure 3 illustrates nine real eigenvalues locate near the unit circle (0.93762, 0.94123, 0.9582, 0.96792, 0.97702, 0.97047, 0.99848, 1.0121, 1.0156), and six eigenvalues are complex conjugate near the unit circle (0.94887 ± 0.052618i, 0.98245 ± 0.0033062i, 1.0516 ± 0.023984i). In other words, fifteen eigenvalues of the interaction matrix *A* locate together at the same region near the unit circle | z | = 1, which demonstrate oscillations in the p53 system with almost the same frequency or period (Ciliberto et al. 2005; Geva-Zatorsky et al. 2006; Kitano, 2004b; Lahav et al. 2004). Four eigenvalues locate a little bit outside the unit circle (1.0121, 1.0156, 1.0516 ± 0.023984i) because time samplings are only from 0 to 12 hours (Barenco et al. 2006) (see Fig. 2), but oscillation periods are about 5.5 hours (Geva-Zatorsky et al. 2006). Inadequate time sampling periods consequently affect measurement of instability, because periodic signals are still in their transient states of ascending or decaying in finite sampling data, and therefore they are considered as unstable signals in a finite period of oscillation signals (Brillinger, 1981). Therefore, based on the above analysis, most of the genes in the multiple loops of p53 are in oscillation with the same frequency. The slight instability is because of the effect of finite data of periodic signals.

### Sensitivity of the gene expression for perturbated environmental stress

The sensitivity of each gene for the change in parameters is considered as the inverse of robustness of gene regulatory networks (Chen et al. 2005; Weinmann, 1991). Environmental stresses induce several cellular responses, and p53 plays the central role that integrates environmental stress and cellular responses (Holcik and Sonenberg, 2005; Vousden and Lu, 2002). Activation of p53 by different stress signals such as DNA damage and oncogene activation can result in many cellular responses including apoptosis, senescence, cell-cycle arrest, survival, DNA repair and genomic stability (Vousden and Lu, 2002). Therefore, we take multiple feedback loops of p53 in Figure 1 as the example of sensitivity analysis under perturbated environmental stress, and calculate sensitivity of the sixteen genes in Figure 1 by microarray data between cancer and normal cells under heat shock, oxidative and endoplasmic reticulum (ER) stress (Murray et al. 2004). Detailed mathematical description and estimated parameters of the sixteen genes under environmental stresses in equation (3) are listed in Table 2 and Supplementary Table 1.

Although cancer conceptually appears to be a robust but fragile system, few computational and quantitative evidences support robustness tradeoffs in the cancer system. To gain objective comparisons between cancer and normal cells, we unify the comparative conditions between cancer and normal cells. First, we only calculate microarray data for 24 hours to reduce biased comparisons caused by different time intervals. Next, we unify perturbations in this study as normal distributed noises with zero mean and 0.1 standard deviations. Moreover, we simulate each interpolated time sampling as 1,000 times and then take the median of these 1,000 sensitivity values because some extreme values will cause deviations. For simplicity in comparisons of these sixteen genes between cancer and normal cells under different stresses, we take the average of this time-dependent sensitivity in equation (6) and list the sensitivity scores in Table 3. Table 3 illustrates comparisons of sensitivity scores among genes in the regulatory network with ranking, where bold and red marks stand for the genes with a higher sensitivity value under each of the three stresses between cancer and normal cells.

### Sensitive genes in multiple feedback loops of p53 between cancer and normal cells

Multiple feedback loops heighten robustness of tumors at intracellular levels, which can result in robustness against chemotherapy (Kitano, 2004b). When MDM2 is degraded to upregulate p53 activity, activated p53 also activates MDM2 which negatively regulates p53 and forms the autoregulatory feedback loop (Geva-Zatorsky et al. 2006). Over-expression of MDM2 in cancer cells causes degradation of p53 and evades apoptosis (Harris and Levine, 2005). In Table 3, p53 and MDM2 are the two genes with high sensitivity scores in normal cells under different stresses, which shows that these diverse cellular stress signals all feed into this p53-MDM2 autoregulatory feedback loop. Transcription factor p53 responds to diverse stresses and regulates many target genes, and MDM2 inhibits p53 in normal conditions (Harris and Levine, 2005; Levine et al. 2006; Toledo and Wahl, 2006). Multiple feedback redundancies of p53 form the bow-tie architecture, which is robust at systems level but fragile on essential elements such as p53 and MDM2 (Kitano, 2007). Some extreme values in normal cells such as PP2A under heat shock, MAPK14 under oxidative stress, and CCNG1 might be triggered fragilely by signal transduction (Harris and Levine, 2005).

Although p53 is not the gene with the highest sensitivity score in cancer cells, MDM2 has extremely high sensitivity score in cancer cells under heat shock, oxidative and ER stress (See Table 3). Since cancer cells lose control abilities under these stresses which result in loss of fidelity in cellular duplication process, P53 and MDM2 proteins are extensively modified after a stress signal between this autoregulatory feedback loop (Geva-Zatorsky et al. 2006; Lahav et al. 2004; Levine et al. 2006). We demonstrate that p53 is activated as the central node in normal cells but not in cancer cells under perturbated environmental stresses, because p53 is mutated about 50% of a wide variety of cancers (Levine et al. 2006). Loss-of-function mutations of p53 in cancer are strongly associated with an increased susceptibility to cancer, and most functions of p53 have been considered in the light of how p53 might help prevent malignant progression (Vousden and Lane, 2007).

### Sensitive genes in multiple feedback loops of p53 under different stresses

Stress signals all affect upon the cellular homeostatic mechanisms that monitor and control the fidelity of DNA replication, chromosome segregation and cell division (Herr and Debatin, 2001). Stresses that activate p53 include damage to the integrity of DNA in a cell, UV irradiation, DNA cross-linking, reaction with oxidative free radicals and heat shock, etc (Levine et al. 2006). Therefore, comparisons of sensitive genes can clearly depict the mechanisms of p53 activation under different stresses. However, we cannot directly compare the value of sensitivity scores under different stresses due to different experimental procedures. Therefore, we compare three genes with the highest sensitivity scores among sixteen genes under three stresses in Table 3.

Since a drug can be effective if it hits the point of fragility (Kitano, 2007), we compare three genes with highest sensitivity scores and find extreme fragility under different stresses. In a cancer cell, the three genes with the highest scores are MDM2, p73, and p21 under heat shock, as well as p73, MDM2 and Siah-1 under oxidative stress, and MDM2, ARF and p73 under ER stress. In a normal cell, the three genes with the highest sensitivity scores are MDM2, p21, and PP2A under heat shock, as well as MAPK14, p53 and MDM2 under oxidative stress and CCNG1, p53 and MDM2 under ER stress (See Table 3). The likeliness between cancer and normal cells is that MDM2 is always the gene with high sensitivity score, but p53 is sensitive only in normal cells compared with p73 in cancer cells. p73 is an example of fragility in the cancer system, since sensitivity of p73 is much higher than other genes in the cancer cell. In other words, p73 is a good drug target by its fragility in cancer but not in normal cells for cancer drug target discovery (Chen and Li, 2007; Kitano, 2007). Since rankings of sensitive genes in under the three stresses are all distinct in Table 3, we can also conclude that different stress signals will induce different responses (Vousden and Lane, 2007).

### Cancer: the robust but fragile system

Cancer is a robust system that prevents apoptosis but also a fragile (or sensitive) system that promotes cell cycle progression and metastasis (Kitano, 2004a, b, 2007; Stelling et al. 2004). In Table 3, sensitivity scores of p53 under heat shock, oxidative and ER stress in normal cell are 0.554, 0.289 and 0.488, which are higher than 0.074, 0.05, 0.046 in cancer cells under the three stresses, respectively. Because p53 is seemed as ‘the guardian of the genome’, sensitivity scores of p53 which are higher in normal cells than in cancer cells indicate cancer is a less sensitive or more robust system. Besides, genes with higher sensitivity scores in normal cells than in cancer cells include p53, MDM2, p21, MAPK14, Wip-1, Siah-1, β-catenin, AKT2, CCNG1, PP2A and p73 under heat shock, p53, ARF, p21, Rb, MAPK14, Wip-1, Siah-1, PIP3, AKT2, CCNG1 and PP2A under oxidative stress, and p53, p21, Rb, Wip-1, β-catenin, PTEN, PIP3, AKT2 and CCNG1 under ER stress (see Table 3 with red and bold marks). In other words, there are more than half of the sixteen genes in feedback loops of p53 with higher sensitivity scores in normal cells under heat shock, oxidative and ER stress. Since p53 and half of the sixteen genes in Figure 1 are less sensitive or more robust in cancer cells than in normal cells under the three stresses, we can conclude that cancer is a ‘more robust’ system compared with normal cell. Hence, we not only demonstrate the robust but fragile property in cancer cell but also clearly illustrate sensitivity and robustness of each gene between cancer and normal cells, which help the identification of cancer drug target discovery (Kitano, 2007).

### Sensitivity of gene expressions to the perturbations in other gene expression

Measurement of the perturbated system △*x̄*[*t* + 1] caused by small input perturbation △*x̄*[*t*] could analyze stability and robustness in equations (7)–(9). Estimated parameter *A* in Table 2 and Supplementary Table 1 determines the stability and robustness of the system, and eigenvalues of *A* listed in Table 4 determine stability in the gene network. According to the system theory, perturbations of some genes will propagate in the gene network if some eigenvalues of the network are near the unit circle.

In Table 4, we can observe that some eigenvalues in both cancer and normal cells under different stresses are near the unit circle. The number of the complex magnitude of eigenvalues ≥0.95 or ≤1.05 in normal cell is 5, 9 and 10, as well as 8, 10 and 11 in cancer cell under heat shock, oxidative and ER stress, respectively. Therefore, we conclude that perturbations of some genes will be kept in the gene circuit network for a long time both in cancer and normal cells. In fact, we use the linear stochastic model in this study, but nonlinearity exists ubiquitously in biological systems. The advantage of the linear model is its simplicity and effectiveness, but it neglects the effects of nonlinearity of the p53 pathway. Therefore, sensitivity of the gene expression to the perturbations in other gene expression in the p53 system indicates the existence of these nonlinear effects in p53 feedback loops, which cannot be interpreted by linear gene network and it needs further analysis.

### Sensitivity of gene expression to changes in kinetic parameters

The sensitivity of gene expression to Δ*A* in equations (10)–(12) could measure how strongly the gene expression is affected by perturbations in kinetic parameters. This method could measure which perturbated parameters cause the system the most serious variations. In other words, we can indicate which genes are the most sensitive to the perturbated parameters by this method. We illustrate our result as the sensitivity scores in cancer cells under heat shock in Supplementary Table 2.

Heat shock results in the phosphorylation of eukaryotic initiator factor 2*α*, inhibition of global translation and induction of stress responses (Holcik and Sonenberg, 2005). Since thermal perturbations by heat shock lead to dramatic changes in kinetic parameters, equations (10)–(12) and Supplementary Table 2 provide useful information in the estimated influence of perturbated kinetic parameters. First, we can estimate degree of these sixteen genes influenced by each perturbated element *α_i,j_* of Δ*A* in equation (10). For example, if we consider the sensitivity of p53 ≥100 in Supplementary Table 2, *α*_1,6_, *α*_3,1_, *α*_6,5_, *α*_11,11_, *α*_12,11_ of △*A_i, j_*, ∀*i*, *j* = 1 … 16, are the five perturbated parameters that cause the gene expression with the most serious deviations in equations of Table 3. We can set up threshold value of the gene which we choose and compare the sensitivity in these perturbated elements. Besides, among sixteen genes in one row of selected perturbated element such as *α*_1,1_ in Supplementary Table 2, we can find that cdk2 and ARF are the two genes with the highest sensitivity scores by Δ*A*_1,1_. The same situation occurs in other perturbated elements of Δ*A*, and we can conclude that cdk2 and ARF are the two genes with the highest sensitivity under perturbations of *A*. Therefore, perturbations of kinetic changes could illustrate which perturbated parameters cause the system the most serious variations, and which genes are the most sensitive to the perturbated parameters.

## Discussion

### Linear and nonlinear model in biological systems

In this study, we apply the linear model to describe multiple feedback loops of p53. Measurement of robustness and sensitivity can be easily calculated by the linear model, but nonlinear effects in the gene network such as DNA-protein complexes are neglected. Robustness is measured by eigenvalue locations, and three types of sensitivity are generated directly by perturbated parameters. Although linear model is easy and efficient to measure robustness and sensitivity compared with the nonlinear model, nonlinear biochemical models such as generalized mass action (GMA) and S-system (Chen et al. 2005; Voit and Ferreira, 2000) should be developed to measure robustness and sensitivity more precisely in future, for example, the oscillation in the linear gene network may correspond to the limit cycle in the nonlinear gene network.

### Robustness-based approach to systems-oriented drug design

Diseases can be viewed as the breakdown and reestablishment of robustness in normal physiological systems, while cancer can be considered as parasitic diseases in which the mechanisms for robustness are hijacked for triggering and progression of the disease state in face of various perturbations including therapeutic interventions (Kitano, 2007). The robustness-based cancer drug design has a specific goal, to find a set of drug targets with specific robustness or sensitivity for which combinatorial perturbation produces both efficacy and selectivity (Chen and Li, 2007).

In Table 3, p53 and MDM2 are two sensitive genes between cancer and normal cells, which make the two attractive drug targets for new therapies (Kitano, 2007). Because p53 can induce tumor cell death and p53 function is lost in most cancers, the most active avenue is to identify small molecules that will allow the reactivation of p53 or block MDM2, such as Nutlin-3 which blocks the interaction of p53/MDM2, and HLI98 which directly targets the ubiquitin-ligase activity of MDM2 (Vousden and Lane, 2007). p73 is also a drug target by its fragility in cancer cells, with similar overall structure to p53 (Vousden and Lane, 2007). If we collect time-dependent microarray data from patients with several types of cancer, we can efficiently compute sensitivity scores by our method, identify possible drug targets, and compare predicted cancer drug targets in different types of cancer. This method can be applied to other gene regulatory network from equations (3)–(12), and combination of these submodule networks is the basis for the robustness-based combinational cancer drug therapy (Chen and Li, 2007; Kitano, 2007).

In Table 3, sensitivity of MDM2 in cancer cells under ER stress is higher than in normal cells, which demonstrates the therapeutic strategy for manipulating the ER stress response. The protein-folding compartment of the endoplasmic reticulum (ER) is particularly sensitive to disturbances, which may trigger apoptosis (Herr and Debatin, 2001). Manipulating the ER stress response of tumor cells is an obtrusive therapeutic strategy, which the ER stress responses involve transcriptional induction of ER chaperones and folding enzymes, translational attenuation to prevent further load of proteins into the ER, and ER-associated degradation to clear misfolded proteins out of the ER (Linder and Shoshan, 2005).

## Conclusion

Robustness describes the persistence of the characteristic behavior of the system under perturbations or conditions of uncertainty, and sensitivity is considered as the inverse of robustness. Oscillations in multiple feedback loops of p53 with almost the same frequency or period are illustrated successfully by locations of eigenvalues, and robustness tradeoffs in cancer are also demonstrated in this study. Besides, we also provide three types of sensitivity measurement, which are useful when estimating system stability and deviations of the system by perturbated parameters under different stresses. These methods provide linear models in robustness-based cancer drug design, and nonlinear models should be considered in future.

## Supplementary Material

**Supplementary Table 1:** Estimated parameters in Table 2 between cancer and normal cells under different stresses.

	Heat shock		Oxidative Stress		ER stress	
	Cancer	Normal	Cancer	Normal	Cancer	Normal
*α* _1,1_	1.044	0.7268	0.9574	0.9616	0.9177	1.074
*α* _1,3_	−0.1026	0.1134	0.006334	0.1298	−0.04867	0.00081
*α* _1,6_	0.05995	0.00317	−0.004416	0.03642	−0.007575	0.1465
*α* _1,7_	0.01031	0.6302	0.04509	0.1503	−0.02275	−0.2139
*α* _2,1_	−0.1564	0.009837	−0.07192	0.008701	0.5371	−0.03559
*α* _2,2_	0.8959	0.9048	0.8932	0.957	1.07	0.8758
*α* _2,10_	−0.1103	−0.003157	0.102	−0.03569	−0.02597	−0.3406
*α* _3,1_	1.745	−0.3639	−0.7279	0.07657	0.2551	0.09949
*α* _3,2_	0.429	2.362	0.8698	−0.2835	0.1324	−0.1144
*α* _3,3_	0.8536	0.6808	0.2286	0.9705	0.8011	0.9916
*α* _3,5_	0.545	−1.227	0.6009	0.01383	0.006959	0.2713
*α* _3,6_	0.3013	−0.1417	0.3345	0.1314	−0.1614	−0.02891
*α* _3,13_	0.775	1.028	−0.1643	0.2014	0.1074	−0.0713
*α* _3,15_	0.4395	0.3443	−0.639	−0.1043	−0.1206	0.1221
*α* _3,16_	0.4395	0.3443	−0.06912	0.1243	0.0601	0.08655
*α* _4,1_	0.04386	−0.4518	−0.02878	−0.05007	−1.154	0.0642
*α* _4,4_	1.007	1.035	0.9609	1.088	0.9964	0.9234
*α* _5,4_	0.02466	−0.00941	−0.04889	0.01455	0.0166	0.01825
*α* _5,5_	0.9195	0.9364	0.9619	1.009	0.8492	1.038
*α* _6,5_	−0.01429	−0.01426	0.01484	−0.03123	0.003516	0.1004
*α* _6,6_	0.9076	0.9634	0.9975	0.9633	0.9813	0.9205
*α* _7,7_	0.9559	0.9505	0.8771	−0.003746	0.9712	0.9715
*α* _7,8_	−0.008813	0.116	−0.03773	0.9206	−0.0272	−0.008996
*α* _8,1_	0.04043	0.08352	−0.06336	−0.06863	−0.4614	−0.03286
*α* _8,8_	1.004	0.95	0.9101	0.9299	0.838	0.9771
*α* _9,1_	0.01675	−0.02692	0.1083	−0.03747	0.2844	0.004906
*α* _9,9_	0.9792	0.9774	0.9507	0.894	0.9698	0 0.9758
*α* _10,9_	−0.008387	0.02753	0.004947	0.003376	−0.009407	−0.05058
*α* _10,10_	0.9849	0.9891	1.034	1.003	1.01	0.8575
*α* _11,1_	0.004463	−0.02747	0.08688	−0.01919	−0.2498	−0.009152
*α* _11,11_	0.9275	0.9473	0.973	0.96	0.9991	0.9859
*α* _12,11_	0.4629	0.4408	0.03193	0.006276	−0.07244	−0.004912
*α* _12,12_	0.4629	0.4408	0.9559	1.006	0.9513	0.9308
*α* _13,12_	0.04349	−0.5637	0.004907	0.002787	0.0072	−0.04064
*α* _13,13_	0.9519	0.8627	1	0.9959	0.9985	0.9816
*α* _14,1_	0.02952	−0.05729	−0.005054	0.01943	0.05795	0.1241
*α* _14,14_	1.009	1.052	1.004	1	0.9891	0.6846
*α* _15,14_	−0.02022	0.01556	−0.03691	−0.05527	0.03189	−0.0311
*α* _15,15_	0.9266	0.8891	0.8629	0.9462	1.004	0.9938
*α* _16,1_	−0.0004636	−0.04371	0.3009	0.0124	0.2117	0.046
*α* _16,13_	−0.005901	−0.0002026	0.103	0.01417	−0.1269	−0.05952
*α* _16,14_	−0.01709	0.05491	0.03214	−0.06075	0.07642	−0.07678
*α* _16,16_	0.924	1.013	1.045	0.937	0.925	0.9677
*β* _1_	0.01208	−0.335	−0.002459	−0.1463	−0.002459	−0.003217
*β* _2_	0.1753	0.05707	0.04106	0.04027	0.04106	0.2366
*β* _3_	−2.499	−1.083	0.2417	−0.08982	0.2417	−0.1813
*β* _4_	−0.01477	0.1958	0.03316	−0.009949	0.03316	0.0329
*β* _5_	0.07958	0.04697	0.04301	−0.01343	0.04301	−0.03471
*β* _6_	0.06151	0.0233	−0.007604	0.03372	−0.007604	0.002424
*β* _7_	0.02485	−0.04106	0.08378	0.04512	0.08378	0.02556
*β* _8_	−0.01578	−0.009031	0.07676	0.07833	0.07676	0.05326
*β* _9_	0.001499	0.02581	−0.03155	0.07065	−0.03155	0.01194
*β* _10_	0.01053	−0.003166	−0.02052	−0.004804	−0.02052	0.0951
*β* _11_	0.03175	0.03484	−0.03126	0.03382	−0.03126	0.01811
*β* _12_	0.03461	0.04657	0.005745	−0.01031	0.005745	0.0334
*β* _13_	0.0001118	0.2914	−0.003158	−0.001051	−0.003158	0.03189
*β* _14_	−0.0173	−0.01331	0.003529	−0.01026	0.003529	0.07618
*β* _15_	0.04294	0.03025	0.09271	0.04081	0.09271	0.02294
*β* _16_	0.04687	−0.02949	−0.2458	0.05101	−0.2458	0.07875

**Supplementary Table 2:** Sensitivity of **Δ*A*** in equations (10)–(12) in cancer cells under heat shock.

Δ *A _i,j_*	p53	ARF	MDM2	p21	cdk2	Rb	MAPK14	Wip-1	Siah-1	β-catenin	PTEN	PIP3	AKT2	CCNG1	PP2A	p73
*α* _1,1_	62.91	104.48	93.86	54.28	151.94	75.73	72.25	96.55	81.12	68.16	93.92	84.44	46.21	73.95	73.92	93.93
*α* _1,3_	54.36	90.28	81.10	46.91	131.29	65.44	62.43	83.43	70.09	58.90	81.16	72.96	39.93	63.90	63.87	81.16
*α* _1,6_	477.88	793.73	713.02	412.38	1154.27	575.33	548.86	733.47	616.23	517.79	713.51	641.44	351.03	561.78	561.52	713.54
*α* _1,7_	91.33	151.70	136.27	78.82	220.61	109.96	104.90	140.18	117.77	98.96	136.37	122.59	67.09	107.37	107.32	136.37
*α* _2,1_	50.17	83.33	74.86	43.30	121.18	60.40	57.62	77.00	64.70	54.36	74.91	67.34	36.85	58.98	58.95	74.91
*α* _2,2_	40.09	66.58	59.81	34.59	96.83	48.26	46.04	61.53	51.69	43.43	59.85	53.81	29.45	47.12	47.10	59.86
*α* _2,10_	55.29	91.84	82.50	47.71	133.55	66.57	63.50	84.86	71.30	59.91	82.55	74.22	40.62	65.00	64.97	82.56
*α* _3,1_	128.60	213.54	191.83	111.45	310.53	154.80	147.68	197.33	165.80	139.32	191.96	172.58	94.61	151.15	151.08	191.97
*α* _3,2_	46.96	77.99	70.05	41.07	113.47	56.52	53.92	72.06	60.54	50.87	70.10	63.01	34.65	55.18	55.16	70.10
*α* _3,3_	55.65	92.26	82.89	48.62	134.14	66.93	63.85	85.27	71.67	60.25	82.95	74.59	41.12	65.35	65.32	82.95
*α* _3,5_	71.99	119.40	107.27	62.76	173.61	86.60	82.62	110.35	92.73	77.96	107.34	96.51	53.14	84.55	84.52	107.34
*α* _3,6_	50.17	83.26	74.79	43.90	121.13	60.36	57.58	76.94	64.65	54.33	74.84	67.28	37.08	58.93	58.91	74.84
*α* _3,13_	61.08	101.34	91.04	53.30	147.35	73.49	70.11	93.65	78.70	66.15	91.10	81.91	45.07	71.75	71.72	91.10
*α* _3,15_	60.96	101.08	90.81	53.18	146.97	73.32	69.95	93.42	78.52	66.01	90.88	81.71	45.01	71.59	71.56	90.88
*α* _3,16_	95.58	158.83	142.65	83.07	231.11	115.08	109.77	146.76	123.27	103.55	142.75	128.31	70.37	112.35	112.31	142.76
*α* _4,1_	44.69	74.23	66.68	38.57	107.95	53.81	51.33	68.60	57.63	48.42	66.73	59.99	32.83	52.54	52.51	66.73
*α* _4,4_	58.94	97.90	87.94	50.86	142.37	70.96	67.70	90.47	76.01	63.86	88.00	79.12	43.30	69.29	69.26	88.01
*α* _5,4_	67.07	111.40	100.07	57.88	162.01	80.75	77.03	102.94	86.49	72.67	100.14	90.03	49.27	78.85	78.81	100.15
*α* _5,5_	74.10	123.07	110.56	63.94	178.98	89.21	85.10	113.73	95.55	80.29	110.63	99.46	54.43	87.11	87.07	110.64
*α* _6,5_	128.80	213.93	192.18	111.15	311.10	155.06	147.93	197.69	166.09	139.56	192.31	172.88	94.61	151.41	151.34	192.32
*α* _6,6_	72.60	120.59	108.33	62.65	175.36	87.41	83.39	111.43	93.62	78.67	108.40	97.45	53.33	85.35	85.31	108.41
*α* _7,7_	76.58	127.19	114.26	66.08	184.96	92.19	87.95	117.53	98.75	82.97	114.34	102.79	56.25	90.02	89.98	114.34
*α* _7,8_	65.35	108.54	97.51	56.39	157.85	78.68	75.06	100.30	84.27	70.81	97.57	87.72	48.00	76.82	76.79	97.58
*α* _8,1_	64.75	107.54	96.61	55.87	156.39	77.95	74.36	99.38	83.49	70.15	96.67	86.91	47.56	76.12	76.08	96.68
*α* _8,8_	34.85	57.88	51.99	30.07	84.17	41.95	40.02	53.48	44.93	37.76	52.03	46.77	25.60	40.96	40.94	52.03
*α* _9,1_	49.96	82.98	74.54	43.11	120.67	60.15	57.38	76.68	64.42	54.13	74.59	67.06	36.70	58.73	58.70	74.60
*α* _9,9_	56.64	94.08	84.52	48.88	136.82	68.20	65.06	86.94	73.04	61.38	84.58	76.03	41.61	66.59	66.56	84.58
*α* _10,9_	60.20	99.98	89.81	51.95	145.40	72.47	69.14	92.39	77.62	65.22	89.88	80.80	44.22	70.76	70.73	89.88
*α* _10,10_	44.18	73.38	65.91	38.12	106.70	53.19	50.74	67.80	56.97	47.87	65.96	59.30	32.45	51.93	51.91	65.96
*α* _11,1_	49.81	82.73	74.31	42.98	120.30	59.96	57.20	76.45	64.23	53.97	74.37	66.85	36.59	58.55	58.52	74.37
*α* _11,11_	153.19	254.45	228.57	132.20	370.02	184.43	175.95	235.13	197.54	165.99	228.73	205.63	112.53	180.09	180.01	228.74
*α* _12,11_	726.80	1207.18	1084.42	627.19	1755.51	875.01	834.75	1115.52	937.21	787.50	1085.17	975.56	533.88	854.41	854.01	1085.22
*α* _12,12_	59.19	98.30	88.31	51.08	142.95	71.25	67.98	90.84	76.32	64.13	88.37	79.44	43.48	69.58	69.54	88.37
*α* _13,12_	46.13	76.62	68.83	39.81	111.42	55.53	52.98	70.80	59.48	49.98	68.87	61.92	33.88	54.23	54.20	68.88
*α* _13,13_	38.01	63.14	56.72	32.80	91.82	45.77	43.66	58.35	49.02	41.19	56.76	51.03	27.92	44.69	44.67	56.76
*α* _14,1_	79.15	131.46	118.09	68.30	191.17	95.28	90.90	121.48	102.06	85.76	118.17	106.23	58.14	93.04	93.00	118.18
*α* _14,14_	44.06	73.18	65.74	38.02	106.42	53.04	50.60	67.63	56.82	47.74	65.79	59.14	32.37	51.80	51.77	65.79
*α* _15,14_	45.47	75.52	67.84	39.24	109.82	54.74	52.22	69.78	58.63	49.26	67.89	61.03	33.40	53.45	53.42	67.89
*α* _15,15_	56.83	94.39	84.79	49.04	137.27	68.42	65.27	87.22	73.28	61.58	84.85	76.28	41.75	66.81	66.78	84.85
*α* _16,1_	96.68	160.58	144.25	83.43	233.53	116.39	111.04	148.39	124.67	104.75	144.35	129.77	71.02	113.65	113.60	144.36
*α* _16,3_	50.50	83.87	75.34	43.58	121.97	60.79	58.00	77.50	65.12	54.71	75.40	67.78	37.09	59.36	59.33	75.40
*α* _16,14_	47.29	78.54	70.56	40.81	114.22	56.93	54.31	72.58	60.98	51.24	70.60	63.47	34.74	55.59	55.56	70.61
*α* _16,16_	88.04	146.23	131.36	75.97	212.65	105.99	101.11	135.12	113.53	95.39	131.45	118.17	64.67	103.50	103.45	131.45

## Figures and Tables

**Table 1:** Ordinary difference equations of sixteen genes in feedback loops of p53.

	Gene name	Mathematical description of sixteen genes in feedback loops of p53
*x1*	p53	*x*1[*t* + 1] = *α*_1,1_*x*1[*t*] + *α*_1,3_*x*3[*t*] + *α*_1,6_*x*6[*t*] + *α*_1,7_*x*7[*t*] + *k*_1_ + *ɛ*_1_
*x2*	ARF	*x*2[*t* + 1] = *α*_2,1_*x*1[*t*] + *α*_2,2_*x*2[*t*] + *α*_2,10_*x*10[*t*] + *k*_2_ + *ɛ*_2_
*x3*	MDM2	*x*3[*t* + 1] = *α*_3,1_*x*1[*t*] + *α*_3,2_*x*2[*t*] + *α*_3,3_*x*3[*t*] + *α*_3,5_*x*5[*t*] + *α*_3,6_*x*6[*t*] + *α*_3,13_*x*13[*t*] + *α*_3,15_*x*15[*t*] + *α*_3,16_*x*16[*t*] + *k*_3_ + *ɛ*_3_
*x4*	p21	*x*4[*t* + 1] = *α*_4,1_*x*1[*t*] + *α*_4,4_*x*4[*t*] + *k*_4_ + *ɛ*_4_
*x5*	cdk2	*x*5[*t* + 1] = *α*_5,4_*x*4[*t*] + *α*_5,5_*x*5[*t*] + *k*_5_ + *ɛ*_5_
*x6*	Rb	*x*6[*t* + 1] = *α*_6,5_*x*5[*t*] + *α*_6,6_*x*6[*t*] + *k*_6_ + *ɛ*_6_
*x7*	MAPK14	*x*7[*t* + 1] = *α*_7,7_*x*7[*t*] + *α*_7,8_*x*8[*t*] + *k*_7_ + *ɛ*_7_
*x8*	Wip-1	*x*8[*t* + 1] = *α*_8,1_*x*1[*t*] + *α*_8,8_*x*8[*t*] + *k*_8_ + *ɛ*_8_
*x9*	Siah-1	*x*9[*t* + 1] = *α*_9,1_*x*1[*t*] + *α*_9,9_*x*9[*t*] + *k*_9_ + *ɛ*_9_
*x10*	β-catenin	*x*10[*t* + 1] = *α*_10,9_*x*9[*t*] + *α*_10,10_*x*10[*t*] + *k*_10_ + *ɛ*_10_
*x11*	PTEN	*x*11[*t* + 1] = *α*_11,1_*x*1[*t*] + *α*_11,11_*x*11[*t*] + *k*_11_ + *ɛ*_11_
*x12*	PIP3	*x*12[*t* + 1] = *α*_12,11_*x*11[*t*] + *α*_12,12_*x*12[*t*] + *k*_12_ + *ɛ*_12_
*x13*	AKT2	*x*13[*t* + 1] = *α*_13,12_*x*12[*t*] + *α*_13,13_*x*13[*t*] + *k*_13_ + *ɛ*_13_
*x14*	CCNG1	*x*14[*t* + 1] = *α*_14,1_*x*1[*t*] + *α*_14,14_*x*14[*t*] + *k*_14_ + *ɛ*_14_
*x15*	PP2A	*x*15[*t* + 1] = *α*_15,14_*x*14[*t*] + *α*_15,15_*x*15[*t*] + *k*_15_ + *ɛ*_15_
*x16*	p73	*x*16[*t* + 1] = *α*_16,1_*x*1[*t*] + *α*_16,3_*x*3[*t*] + *α*_16,14_*x*14[*t*] + *α*_16,16_*x*16[*t*] + *k*_16_ + *ɛ*_16_

*α*_1,1_ = 0.9316; *α*_1,3_ = −0.4334; *α*_1,6_ = −0.02982; *α*_1,7_ = 0.1688; *α*_2,1_ = 0.003243; *α*_2,2_ = 0.9704; *α*_2,10_ = −0.002874; *α*_3,1_ = −0.0314; *α*_3,2_ = −0.09199; *α*_3,3_ = 0.8198; *α*_3,5_ = 0.05164; *α*_3,6_ = −0.1261; *α*_3,13_ = 0.3148; *α*_3,15_ = 0.07828; *α*_3,16_ = −0.08918; *α*_4,1_ = 0.02437; *α*_4,4_ = 09695; *α*_5,4_ = −0.02353; *α*_5,5_ = 1.054; *α*_6,5_ = 0.106; *α*_6,6_ = 0.9907; *α*_7,7_ = 0.9733; *α*_7,8_ = 0.01645; *α*_8,1_ = 0.05625; *α*_8,8_ = 0.9581; *α*_9,1_ = 0.01594; *α*_9,9_ = 1.015; *α*_10,9_ = 0.02788; *α*_10,10_ = 0.9582; *α*_11,1_ = 0.000767; *α*_11,11_ = 1; *α*_12,11_ = 0.03649; *α*_12,12_ = 1.009; *α*_13,12_ = 0.06556; *α*_13,13_ = 0.935; *α*_14,1_ = 0.05642; *α*_14,14_ = 0.9832; *α*_15,4_ = 0.001754; *α*_15,15_ = 0.9765; *α*_16,1_ = −0.001995; *α*_16,3_ = 0.02613; *α*_16,14_ = −0.00162; *α*_16,16_ = 0.9525.

**Table 2:** Ordinary difference equations of sixteen genes in feedback loops of p53 under stresses. Estimated parameters are in Supplementary Table 1.

	Gene name	Mathematical description of sixteen genes under stresses
*x1*	p53	*x*1[*t* + 1] = *α*_1,1_*x*1[*t*] + *α*_1,3_*x*3[*t*] + *α*_1,6_*x*6[*t*] + *α*_1,7_*x*7[*t*] + *β*_1_*u*[*t* − 1] + *k*_1_ + *ɛ*_1_
*x2*	ARF	*x*2[*t* + 1] = *α*_2,1_*x*1[*t*] + *α*_2,2_*x*2[*t*] + *α*_2,10_*x*10[*t*] + *β*_2_*u*[*t* − 1] + *k*_2_ + *ɛ*_2_
*x3*	MDM2	*x*3[*t* + 1] = *α*_3,1_*x*1[*t*] + *α*_3,2_*x*2[*t*] + *α*_3,3_*x*3[*t*] + *α*_3,5_*x*5[*t*] + *α*_3,6_*x*6[*t*] + *α*_3,13_*x*13[*t*] + *α*_3,15_*x*15[*t*] + *α*_3,16_*x*16[*t*] + *β*_3_*u*[*t* − 1] + *k*_3_ + *ɛ*_3_
*x4*	p21	*x*4[*t* + 1] = *α*_4,1_*x*1[*t*] + *α*_4,4_*x*4[*t*] + *β*_4_*u*[*t* − 1] + *k*_4_ + *ɛ*_4_
*x5*	cdk2	*x*5[*t* + 1] = *α*_5,4_*x*4[*t*] + *α*_5,5_*x*5[*t*] + *β*_5_*u*[*t* − 1] + *k*_5_ + *ɛ*_5_
*x6*	Rb	*x*6[*t* + 1] = *α*_6,5_*x*5[*t*] + *α*_6,6_*x*6[*t*] + *β*_6_*u*[*t* − 1] + *k*_6_ + *ɛ*_6_
*x7*	MAPK14	*x*7[*t* + 1] = *α*_7,7_*x*7[*t*] + *α*_7,8_*x*8[*t*] + *β*_7_*u*[*t* − 1] + *k*_7_ + *ɛ*_7_
*x8*	Wip-1	*x*8[*t* + 1] = *α*_8,1_*x*1[*t*] + *α*_8,8_*x*8[*t*] + *β*_8_*u*[*t* − 1] + *k*_8_ + *ɛ*_8_
*x9*	Siah-1	*x*9[*t* + 1] = *α*_9,1_*x*1[*t*] + *α*_9,9_*x*9[*t*] + *β*_9_*u*[*t* − 1] + *k*_9_ + *ɛ*_9_
*x10*	β-catenin	*x*10[*t* + 1] = *α*_10,9_*x*9[*t*] + *α*_10,10_*x*10[*t*] + *β*_10_*u*[*t* − 1] + *k*_10_ + *ɛ*_10_
*x11*	PTEN	*x*11[*t* + 1] = *α*_11,1_*x*1[*t*] + *α*_11,11_*x*11[*t*] + *β*_11_*u*[*t* − 1] + *k*_11_ + *ɛ*_11_
*x12*	PIP3	*x*12[*t* + 1] = *α*_12,11_*x*11[*t*] + *α*_12,12_*x*12[*t*] + *β*_12_*u*[*t* − 1] + *k*_12_ + *ɛ*_12_
*x13*	AKT2	*x*13[*t* + 1] = *α*_13,12_*x*12[*t*] + *α*_13,13_*x*13[*t*] + *β*_13_*u*[*t* − 1] + *k*_13_ + *ɛ*_13_
*x14*	CCNG1	*x*14[*t* + 1] = *α*_14,1_*x*1[*t*] + *α*_14,14_*x*14[*t*] + *β*_14_*u*[*t* − 1] + *k*_14_ + *ɛ*_14_
*x15*	PP2A	*x*15[*t* + 1] = *α*_15,14_*x*14[*t*] + *α*_15,15_*x*15[*t*] + *β*_15_*u*[*t* − 1] + *k*_15_ + *ɛ*_15_
*x16*	p73	*x*16[*t* + 1] = *α*_16,1_*x*1[*t*] + *α*_16,3_*x*3[*t*] + *α*_16,14_*x*14[*t*] + *α*_16,16_*x*16[*t*] + *β*_16_*u*[*t* − 1] + *k*_16_ + *ɛ*_16_

**Table 3:** Sensitivity scores under heat shock, oxidative and ER stress between cancer and normal cells. This table provides a snapshot of sensitivity scores with ranking in each condition, where red and bold marks denote each gene with higher sensitivity value between cancer and normal cells under one of the three stresses.

Stresses	Heat shock		Oxidative stress		ER stress	
Cell type Gene name	Cancer	Normal	Cancer	Normal	Cancer	Normal
p53	0.074 (12)	**0.554** (7)	0.05 (12)	**0.289** (2)	0.046 (14)	**0.488** (2)
ARF	**0.228** (6)	0.089 (12)	0.063 (10)	**0.092** (11)	**0.509** (2)	0.358 (5)
MDM2	5.649 (1)	**6.482** (1)	**0.288** (2)	0.252 (3)	**5.568** (1)	0.432 (3)
p21	0.284 (3)	**0.836** (3)	0.107 (6)	**0.148** (7)	0.263 (5)	**0.376 (4)**
cdk2	**0.234** (5)	0.118 (10)	**0.09** (9)	0.052 (14)	**0.351** (4)	0.126 (13)
Rb	**0.123** (8)	0.097 (11)	0.04 (13)	**0.151** (6)	0.075 (11)	**0.154** (11)
MAPK14	0.048 (13)	**0.16** (9)	0.101 (7)	**2.937** (1)	**0.162** (8)	0.076 (15)
Wip-1	0.167 (7)	**0.198** (8)	0.111 (5)	**0.213** (5)	0.187 (7)	**0.326** (6)
Siah-1	0.03 (15)	**0.085** (13)	0.126 (3)	**0.215** (4)	**0.086** (10)	0.053 (16)
β-catenin	0.019 (16)	**0.029** (16)	0.038 (14)	0.038 (16)	0.072 (12)	**0.189** (9)
PTEN	**0.114** (10)	0.08 (14)	**0.092** (8)	0.082 (12)	0.068 (13)	**0.095** (14)
PIP3	**0.118** (9)	0.07 (15)	0.024 (15)	**0.094** (10)	0.029 (15)	**0.308** (7)
AKT2	0.283 (4)	**0.751** (4)	0.011 (16)	**0.044** (15)	0.016 (16)	**0.13** (12)
CCNG1	0.046 (14)	**0.59** (6)	0.057 (11)	**0.061** (13)	0.088 (9)	**1.062** (1)
PP2A	0.091 (11)	**1.214** (2)	0.114 (4)	**0.133** (8)	**0.196** (6)	0.18 (10)
p73	0.291 (2)	**0.738** (5)	**1.1** (1)	0.112 (9)	**0.506** (3)	0.27 (8)

**Table 4:** Eigenvalues of *A* in equation (3) under heat shock, oxidative and ER stress between cancer and normal cells.

	Heat shock	Oxidative stress	ER stress
**Cancer cells**	0.92783 + 0.41426i	0.24283	0.7943
	0.92783 − 0.41426i	0.86287	0.96309 + 0.11392i
	0.46283	0.88826 + 0.035308i	0.96309 − 0.11392i
	1.0111	0.88826 − 0.035308i	0.8439
	1.0047	0.89148	0.86648
	1.0037	1.0348 + 0.001955i	0.92144
	0.98273 + 0.0087054i	1.0348 − 0.001955i	0.95681
	0.98273 − 0.0087054i	1.0038	1.0124 + 0.0079295i
	0.94724 + 0.010911i	0.99953	1.0124 − 0.0079295i
	0.94724 − 0.010911i	0.99684	0.99941 + 0.016265i
	0.95597	0.95284 + 0.015758i	0.99941 − 0.016265i
	0.90653	0.95284 − 0.015758i	0.96999
	0.92026 + 0.0021752i	0.98335	0.98323 + 0.0024292i
	0.92026 − 0.0021752i	0.97226	0.98323 − 0.0024292i
	0.92632 + 0.00091094i	0.9515	1.0061
	0.92632 − 0.00091094i	0.956	0.99636
**Normal cells**	0.48721	−0.014164	0.68463
	0.74185 + 0.22662i	1.0906	1.084
	0.74185 − 0.22662i	0.92727 + 0.057957i	0.9792 + 0.070673i
	0.64244	0.92727 − 0.057957i	0.9792 − 0.070673i
	1.0624 + 0.039692i	0.89009	0.8574
	1.0624 − 0.039692i	0.90593	0.87611
	1.0435	1.0257 + 0.023522i	0.89497
	1.0114	1.0257 − 0.023522i	1.0089 + 0.024866i
	0.88929	0.94764	1.0089 − 0.024866i
	0.91228 + 0.0040725i	0.96265 + 0.003333i	0.9308
	0.91228 − 0.0040725i	0.96265 − 0.003333i	0.95419 + 0.017276i
	0.98871	0.95992	0.95419 − 0.017276i
	0.97765	0.99502	0.99376
	0.93514	1.0066	0.9859
	0.96344	1.0041	0.9816
	0.94719	1.0008	0.9758
